# Hybrid Local Fibers for Enhancing the Mechanical Properties of Engineered Cementitious Composites

**DOI:** 10.3390/ma19132908

**Published:** 2026-07-07

**Authors:** Xiaoyu Qiu, Lina Tang, Yucheng Shi, Hedong Li, Tao Wang

**Affiliations:** 1 Guangdong Provincial Key Laboratory of Durability for Marine Civil Engineering, Shenzhen University, Shenzhen 518060, China; 2453214018@mails.szu.edu.cn; 2 Zhejiang Zheneng Technology & Environment Group Co., Ltd., Hangzhou 311121, China; tanglina@zjenergy.com.cn; 3 School of Civil Engineering and Architecture, Zhejiang Sci-Tech University, Hangzhou 310018, China; 202030702038@mails.zstu.edu.cn; 4 State Key Laboratory of Clean Energy Utilization, Zhejiang University, Hangzhou 310012, China; oatgnaw@zju.edu.cn

**Keywords:** engineered cementitious composite, hybrid local fibers, single-fiber pullout behavior, mechanical properties

## Abstract

Engineered cementitious composites (ECCs) reinforced with imported polyvinyl alcohol (PVA) or polyethylene (PE) fibers exhibit high tensile deformability, but the fiber cost limits the wider application of ECCs. In this study, locally produced PVA and PE fibers were used to develop lower-cost ECC, and PVA–PE fiber hybridization was adopted to improve tensile deformability. Based on matrices with various fly ash volumes, the single-fiber pullout behavior was first investigated at the micromechanical level. The results showed that PVA and PE fibers failed mainly by rupture and pullout, respectively, and that the chemical bonding between PVA fibers and the surrounding matrix decreased with increasing fly ash volume. The effects of single-fiber addition and hybrid-fiber addition on the macromechanical properties of ECC were then examined. The results indicated that ECC reinforced with hybrid PVA–PE fibers exhibited enhanced tensile performance compared with ECC reinforced with either PVA or PE fibers alone, with an ultimate tensile strain exceeding 5.3%, an average crack width below 39 μm, and hybrid reinforcing effect coefficients of 1.17–1.30, indicating a positive hybrid effect. Overall, the lower-cost ECC incorporating hybrid local fibers developed in this study demonstrates promising tensile deformability and crack-control capacity.

## 1. Introduction

Engineered cementitious composites (ECCs) exhibit high tensile ductility by incorporating random short fibers, which changes the brittleness of concrete [1]. Under uniaxial tensile loading, ECC cracking is accompanied by strain hardening and multiple fine cracks, with crack widths typically below 100 μm [2,3]. Owing to these unique characteristics, ECCs have attracted considerable attention in engineering applications, including structural strengthening [4], repair of deteriorated concrete structures [5], and infrastructure rehabilitation [6], where high ductility and effective crack control are particularly desirable. Since fibers play a dominant role in governing crack development and deformation behavior, their properties are critical to the overall performance of ECC. Hydrophilic polyvinyl alcohol (PVA) fibers have been widely used in previous studies because they readily form chemical bonds with the cementitious matrix and help control crack splitting. However, excessive interfacial bonding may cause premature fiber rupture during fiber–matrix separation, thereby reducing the fiber-bridging capacity [7,8,9]. Therefore, researchers have attempted to optimize the PVA–matrix interface, such as fiber surface modification [10] and tailoring of fly ash content [11], endowing better fiber-bridging capacity as well as contributing to the strain-hardening behaviors of ECC. Polyethylene (PE) fibers have also been used in ECC, and previous studies have reported an ultimate tensile strain of nearly 8% or higher [1,12,13,14]. However, PE fibers are several times more expensive than PVA fibers, which increases the material cost of ECC [15,16]. Among the two fiber types mentioned above, the PVA fibers most commonly used in ECC are produced by Kuraray Co. Ltd. in Japan [17,18], whereas PE fibers with high tensile strength and elastic modulus, such as Spectra^®^ 1000 from Honeywell, are generally preferred [19]. The use of imported fibers increases both fiber price and transportation cost, thereby limiting the localized application of ECC. Therefore, the development of cost-effective ECC incorporating locally available fibers is essential for its wider local application. For example, the locally produced PVA fiber costs approximately US$8/kg, which is only about one-quarter to one-third of the price of imported PVA fiber (approximately US$28/kg).

Currently, the tensile strength and elastic modulus of local PVA/PE fibers are generally lower than those of imported fibers, which is theoretically not conducive to fiber-bridging ability and may make it more difficult to satisfy the criteria of steady state crack propagation [20]. In previous research, Kan et al. [20] investigated the performance difference between local and imported PVA-ECC, and the results indicated that although the fiber-bridging capacity and pseudo-strain-hardening index of local PVA-ECC were lower than those of imported PVA-ECC, its tensile strain still exceeded 3%. Wang et al. [21] developed a low-cost ECC using locally produced unoiled PVA fibers and achieved a tensile strain of 1.96%. Yu et al. [22] reported that the tensile strain of ECC incorporating PE fibers (provided by Shandong Aidi Co., Ltd., China) reached up to 6–9% by combining defective particles, such as crumb rubber and hollow fly ash cenosphere. Zhu et al. [1] also prepared ECC using locally produced PE fibers and achieved a tensile strain of 7.35%. Overall, ECC reinforced with locally produced PVA/PE fibers generally exhibits slightly lower deformability than ECC reinforced with imported fibers; nevertheless, it can still achieve the essential features of ECC, including strain hardening and multiple cracking.

Although previous studies have demonstrated the feasibility of producing ECC using either local PVA fibers or local PE fibers, most investigations have focused on single-fiber systems. Compared with single-fiber systems, the combination of local PVA fibers and PE fibers may offer complementary advantages for improving the deformation capacity of ECC while reducing material costs. The specific advantages are as follows: (1) the hydrophilic nature of PVA fibers promotes chemical bonding with the matrix, which facilitates stress transfer across cracks. However, excessive interfacial bonding may lead to premature fiber rupture during pullout. In contrast, the high elastic modulus of PE fibers favors sustained fiber bridging and helps mitigate this limitation. (2) The chemical bonding between hydrophilic PVA fibers and the matrix tends to increase with continued hydration, which is unfavorable for maintaining long-term deformability [23]. In contrast, hydrophobic PE fibers exhibit negligible chemical bonding with the matrix and are therefore less susceptible to hydration-induced interfacial changes. (3) The market price of local PE fiber (approximately US$25/kg) is still 3–4 times that of local PVA fiber (approximately US$8/kg). Consequently, the hybrid use of PVA and PE fibers offers a promising strategy for reducing the overall fiber cost. In addition, although high-volume fly ash has been widely adopted to tailor matrix properties, further investigation is still needed to clarify its combined effect with hybrid local fibers on ECC performance.

This study aimed to develop a lower-cost ECC using local fibers, and the hybrid use of PVA and PE fibers was employed for excellent tensile properties. First, the single-fiber pullout behavior was investigated in terms of micromechanical parameters based on multiple fly ash contents. Then, the macromechanical properties of ECC reinforced with hybrid PVA–PE fibers, including tensile properties and compressive strength, were investigated and compared with those of ECC reinforced with the same total volume of either PVA or PE fibers alone. In addition, different PVA/PE fiber ratios were considered. Finally, the effects of hybrid fibers on mechanical properties and progressive cracking were further discussed using the hybrid reinforcing effect coefficient.

## 2. Experiment Program

### 2.1. Raw Materials

**Figure 1:**
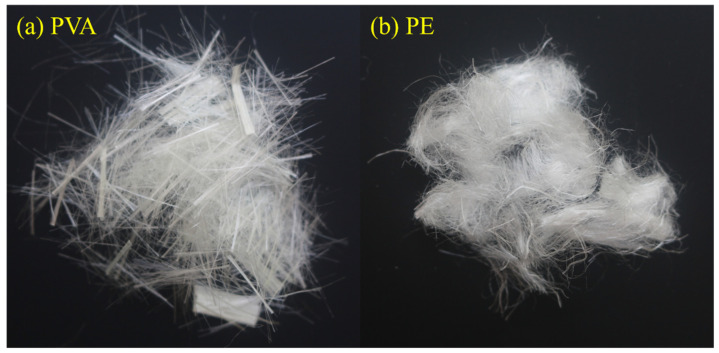
Morphologies of local PVA and PE fibers.

The raw materials used in this study included binders, fine aggregate, water, admixtures, and fibers. The binder materials consisted of C42.5 high-belite sulphoaluminate cement (HBSAC), provided by Tangshan Polar Bear Building Materials Co., Ltd., Tangshan, China, which is characterized by rapid setting and hardening; Class F fly ash (FA); and Elkem 920U Silica fume (SF). SF was incorporated as a supplementary cementitious material to improve matrix compactness and interfacial properties. The chemical compositions of HBSAC and FA are shown in Table 1. The silica sand (SS, 100–200 mesh) was used as the fine aggregate, and tap water was employed. SS was employed to improve particle packing and dimensional stability in the ECC matrix. The admixtures included VINNAPSA^®^ 5010 redispersible emulsion powder (REP); OPTIBENT^®^ 987 thixotropic agent (TA); Boric acid (BA); AE-10 air-entraining agent (AEA); and Melflux 4930F polycarboxylate superplasticizer (SP). The PVA and PE fibers were supplied by different local fiber manufacturers. The PVA fibers used in this study had a diameter of 39 μm and a length of 12 mm, whereas the PE fibers had a diameter of 24 μm and a length of 12 mm, as shown in Figure 1. Their detailed physical and mechanical properties are presented in Table 2.

### 2.2. Mix Proportions and Sample Preparation

As shown in Table 3, ECC mixtures were prepared using matrices containing either 30% or 50% fly ash, with different PVA/PE fiber volume ratios of 2.0/0, 1.5/0.5, 1.0/1.0, and 0/2.0, respectively. Based on the market prices of the locally produced fibers used in this study, the fiber-related material cost of ECC reinforced with 1.5% PVA + 0.5% PE and 1.0% PVA + 1.0% PE was approximately 51% and 34% lower, respectively, than that of ECC reinforced solely with 2% PE fibers.

The matrix and ECC mixtures were prepared as follows:

(1) First, all dry powders except SP were mixed for approximately 2–3 min in a 12 L Hobart mixer to achieve a homogeneous blend.
(2) Second, half of the tap water was added to the dry mixture and stirred for 30 s, and the remaining water and SP were then added and stirred for another 30 s.
(3) Third, fibers were gradually incorporated into several batches, with continuous stirring for approximately 5 min. This step was omitted for the matrix samples.
(4) Finally, the fresh mixtures were poured into molds and covered with plastic film after vibration. The samples were demolded after 24 h and then transferred to a standard curing environment at 20 ± 2 °C and a relative humidity exceeding 95% for 27 days.

The single-fiber pullout samples were cast using a specially designed mold according to the Chinese patent entitled Straight Short-Cut Fiber Pulling Test Specimen Forming Mold and its production method [24]. The fiber embedment length was 2 mm.

### 2.3. Test Procedures

#### 2.3.1. Single-Fiber Pullout Test

**Figure 2:**
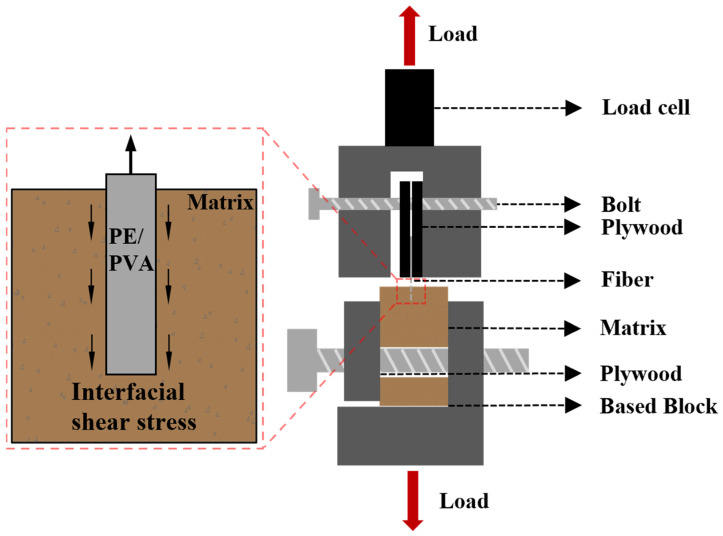
Single-fiber pullout test setup.

**Figure 3:**
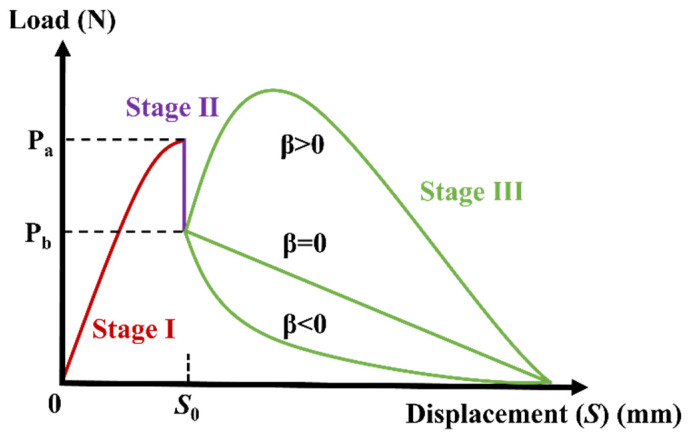
Schematic load–displacement response of single-fiber pullout, developed based on experimental observations obtained in this study and a generalized fiber pullout framework reported in Refs. [27,29].

Figure 2 shows the single-fiber pullout test setup. The matrix portion of each specimen was clamped by tightening a bolt, and a similar method was used to secure the fiber. A base block with the same thickness was used to ensure that the matrix remained perpendicular to the fiber pullout direction. Six samples with single fiber embedment were prepared for each group. The test was conducted using a microcomputer-controlled material testing machine at a loading rate of 0.2 mm/min. A schematic model illustrating the single-fiber pullout behavior is presented in Figure 3. Three pullout parameters, including chemical debonding energy (*G*_d_), frictional bond strength (*τ*_0_), and slip-hardening coefficient (*β*), were determined using Equations (1)–(3) [25].

Unlike PE fibers, PVA fibers form strong chemical bonds with the matrix because of the hydroxyl groups in their molecular chains [26], and this chemical bonding must be overcome during pullout. The entire extraction process can be divided into the following three stages [27]:

(1) Stage I (debonding stage): The chemical bonding between the fiber and matrix initiates to fracture when the load increases from 0 to *P*_a_. Fiber rupture may occur if the load exceeds the tensile capacity of the fiber.
(2) Stage II (full-debonding stage): The chemical bonding is completely broken when the load decreases abruptly from *P*_a_ to *P*_b_, after which fiber slippage begins in the matrix channel. By contrast, PE fibers do not undergo a distinct chemical debonding process because of their hydrophobicity. The determination of *G*_d_ and *τ*_0_ follows Equations (1) and (2), respectively.
  (1) $$G_{d}=\frac{2{\left(P_{a}-P_{b}\right)}^{2}}{\pi^{2}E_{f}d_{f}^{3}}$$
  where *G*_d_ represents the chemical debonding energy; *P*_a_ represents the maximum load for chemical debonding of fiber; *P*_b_ represents the load after the chemical debonding plunge; and *E_f_* and *d_f_* represent the elastic modulus and diameter of fiber, respectively.
  (2) $$\tau_{0}=\frac{P_{b}}{\pi d_{f}L_{e}}$$
  where *τ*_0_ represents the frictional bond strength, and *L*_e_ represents the fiber embedment length.
(3) Stage III (slipping stage): Fiber slippage occurs in the matrix channel at this stage. Since the hardness of PVA fiber is less than that of the surrounding matrix, the fiber’s surface is damaged, and a jamming effect occurs in the matrix channel. Consequently, the pullout load increases further, i.e., slipping hardening (*β* > 0), and the calculation of the slip-hardening coefficient references Equation (3). In contrast, PE fibers are harder than the surrounding matrix, and constant friction (*β* = 0) or slip softening (*β* < 0) may occur [28].
  (3) $$\beta =(d_{f}/L_{e})[(1/\tau_{0}\pi d_{f})(\Delta P/\Delta S{)}_{丨s\to s_{0}}+1]$$
  where Δ*P*/Δ*S* represents the tangent slope of the initial slip period of the fiber.

#### 2.3.2. Uniaxial Tension Test

**Figure 4:**
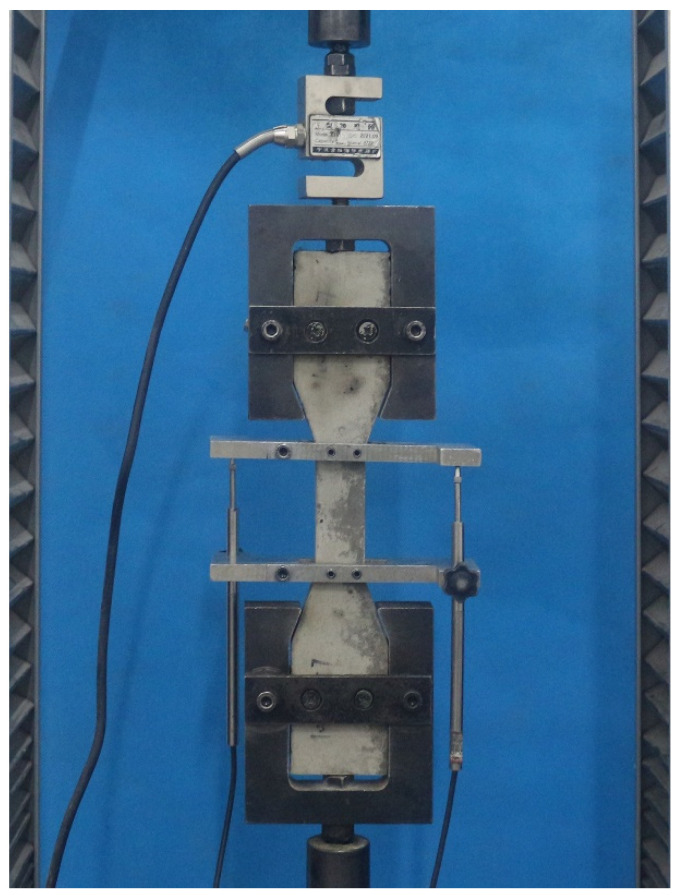
Uniaxial tension test setup.

Four dog bone-shaped tensile samples were prepared for each group. The uniaxial tension test was conducted using a microcomputer-controlled material testing machine at a loading rate of 0.5 mm/min. The test setup of uniaxial tension is shown in Figure 4. According to JC/T 2461-2018 (China) [30], the determination of ultimate tensile strength and ultimate tensile strain was in accordance with Equations (4) and (5). Furthermore, the average number of cracks on the failed surfaces was measured using a microscope, and the average crack width was calculated using Equation (6).

(4) $$f_{\mathrm{tu}}=\frac{F_{\mathrm{tu}}}{A_{t}}$$

where *f*_tu_ means the ultimate tensile strength; *F*_tu_ means the maximum tensile load; and *A*_t_ means the initial cross-sectional area of the deformation measurement region.

(5) $$\varepsilon_{\mathrm{tu}}=\frac{l_{\mathrm{tu}}-l_{0}}{L_{\mathrm{tg}}}\times 100\%$$

where *ε*_tu_ means the ultimate tensile strain; *l*_tu_ means the deformation at maximum load; *l*_0_ means the deformation before the loading; and *L*_tg_ means the gauge length.

(6) $$w_{a}=\frac{\varepsilon_{\mathrm{tu}}L_{\mathrm{tg}}}{N_{c}}\times 1000$$

where *w*_a_ means the average crack width, and *N*_c_ means the number of cracks.

#### 2.3.3. Compression Test

Three 70.7 mm × 70.7 mm × 70.7 mm cubic samples were prepared for each group. The compression test was carried out in accordance with JGJ/T 70-2009 (China) [31] at a loading rate of 0.3 MPa/s.

## 3. Results and Discussion

### 3.1. Single-Fiber Pullout Behaviors

**Figure 5:**
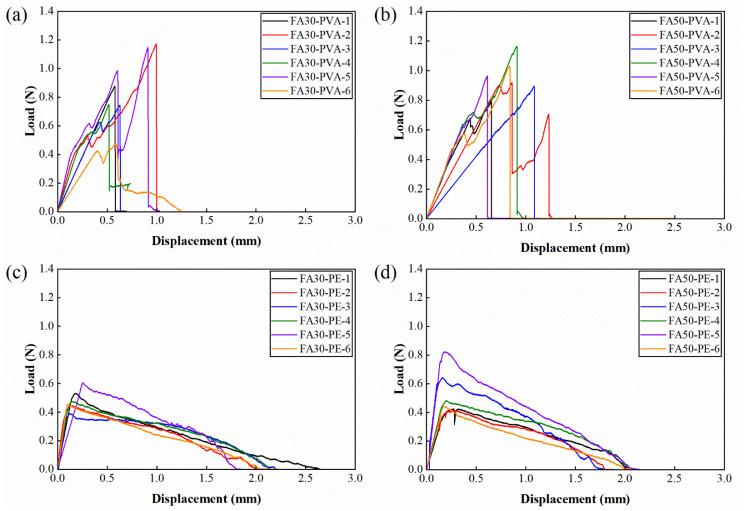
Load–displacement curves of single-fiber pullout: (**a**) FA30-PVA; (**b**) FA50-PVA; (**c**) FA30-PE; (**d**) FA50-PE. Note: FA30-PVA represents the group of single PVA fiber embedded in a matrix of 30% FA, and the same naming convention applies to the other groups.

Figure 5 and Table 4 illustrate the load–displacement curves and the corresponding parameters of single-fiber pullout. As can be seen from the curve morphology, the pullout process of PVA fiber from the matrix went through the debonding stage and the slip hardening derived by increasing interfacial friction during fiber slipping [32], while the PE fiber exhibited slip softening, i.e., a gradual downward trend after reaching the peak load. This phenomenon is attributed to the fact that PVA fiber exhibits a much lower elastic modulus than PE fiber, as shown in Table 2, meaning that PVA fiber is more susceptible to the jamming effect caused by the surrounding matrix during the slippage [27]. Additionally, the hydrophilic nature of PVA fiber tends to form a strong interface bonding with the matrix, while the chemical bonding between PE fiber and matrix is close to zero due to PE fiber’s hydrophobic nature [33]. Therefore, the maximum displacement of the PVA fiber pullout curve was less than the embedding length, indicating that its rupture failure occurred during the pullout procedure, whereas the PE fiber showed almost total pullout failure and could be inferred from the final displacement of the PE fiber pullout curve reaching 2 mm.

In Table 4, comparing the results of FA30-PVA and FA30-PE, FA50-PVA and FA50-PE, the peak load and corresponding displacement of PVA fiber were greater than those of PE fiber, attributed to slip hardening of PVA fiber, which is conducive to the stress conductivity and increase. In the case of using the same fiber type, increasing the fly ash volume contributed to improving the pullout resistance and displacement capacity, with increases of 43.1% and 20% in displacements for PVA and PE fibers in particular when the fly ash content was up to 50% from 30%. The reason for the above phenomenon may be that the spherical geometry of fly ash helps to mitigate the injury to the fibers during the pullout procedure and protect them from premature rupture failure. The parameters of single-fiber pullout indicated that the value of *G*_d_ was 0.61 J/m^2^ and 0.43 J/m^2^, *τ*_0_ was 2.12 MPa and 2.43 MPa, and *β* was 0.11 and 0.1 for FA30-PVA and FA50-PVA, respectively. It can be seen that the increasing addition of fly ash weakened the chemical debonding energy between the PVA fiber and the matrix. This phenomenon occurs because the higher percentage of fly ash diminishes the density of Al^3+^ and Ca^2+^ in the matrix, thereby decreasing the formation of strong chemical bonds [11,34]. Conversely, the frictional bond strength tended to increase with fly ash content. This may be attributed to the spherical morphology and fine particle size of fly ash, which improve particle distribution and packing density around the fiber–matrix interface, thereby potentially enhancing the interfacial contact quality and frictional resistance during fiber pullout as suggested in previous studies [35]. Similar observations have also been reported in previous microstructural studies, where fly ash incorporation was found to modify the fiber–matrix interfacial region and alter the interfacial bonding characteristics, which may contribute to improved fiber pullout behavior [34,35]. From the perspective of ECC design, the measured pullout parameters provide important insights into the optimization of fiber–matrix interactions. The reduction in chemical bonding, accompanied by a moderate increase in frictional bond strength, may favor fiber pullout and sustained fiber bridging, thereby promoting multiple cracking and tensile deformability. These findings help explain the improved mechanical performance observed in ECC incorporating hybrid local fibers and provide guidance for tailoring matrix composition through fly ash incorporation.

### 3.2. Tensile Properties

#### 3.2.1. Tensile Stress–Strain Curves and Tensile Parameters

**Figure 6:**
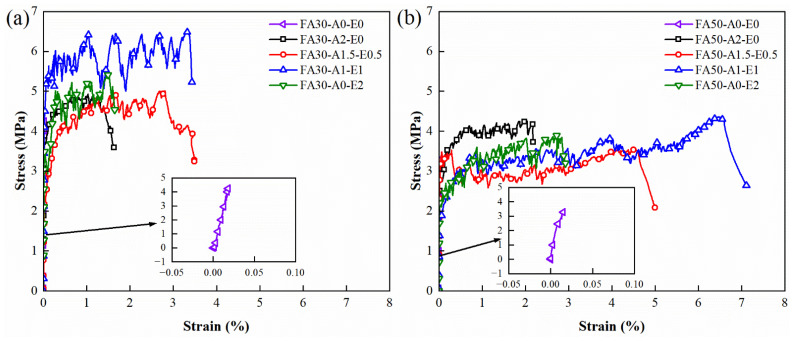
Tensile stress–strain curves of ECCs and matrices: (**a**) FA30 groups; (**b**) FA50 groups.

Figure 6 and Table 5 illustrate the tensile stress–strain curves and tensile parameters of ECCs and corresponding matrices, respectively. Compared to matrix groups, ECCs all exhibited strain-hardening characteristics under uniaxial tension. The experimental results showed that the ultimate tensile strength of FA30-A0-E0 and FA50-A0-E0 was 4.0 MPa and 3.4 MPa, and their ultimate tensile strain was 0.017% and 0.014%, respectively, indicating that a decrease in tensile strength occurred with an increase in fly ash volume. There are several reasons for the effects of fly ash incorporation on the strength: (1) Fly ash possesses a pozzolanic effect, generating hydration products by combining with calcium hydroxide, which is conducive to strength development [36]; (2) the filling effect of fine fly ash particles may improve the internal microstructure, which is likewise beneficial for load transfer [37]; and (3) a reduction in the content of hydrated raw materials will take place when the proportion of fly ash incorporated is increased. The positive benefits of factors (1) and (2) on strength are lower than the negative effects caused by factor (3) when the amount of fly ash increased from 30% to 50%, and that is why the aforementioned reduction in strength happens. Furthermore, the tensile strain capacity of ECCs was 65 to 407 times higher than that of the pure matrix in this study. Such a significant enhancement in tensile deformability is consistent with previous investigations on ECC reported in the literature [38,39].

**Figure 7:**
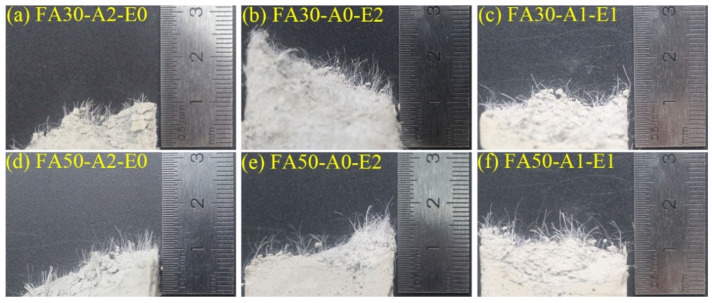
Fiber protrusion at fracture cross-sections.

Comparing the tensile properties of ECC incorporating sole PVA/PE fibers, the ultimate tensile strength and ultimate tensile strain of ECC with sole PE fibers performed better than those with sole PVA fibers, attributed to the higher elastic modulus of PE fiber. Nevertheless, the condition changed with the increase in fly ash addition, and the tensile properties of ECC added with PVA fibers alone were almost close to those of PE fibers alone when fly ash addition reached 50%. The reasons contributing to the above phenomenon are as follows: (1) The excessive interface bonding between PVA fibers and matrix is prone to fiber rupture, and such an adverse situation will be rectified by the increasing percentage of fly ash. According to the results in Section 3.1, the *G*_d_ between PVA fiber and matrix reduced when the fly ash volume increased from 30% to 50%. Meanwhile, we had observed that the protrusion of fibers at the fracture cross-section of FA50-A2-E0 was longer compared to FA30-A2-E0, as can be seen in Figure 7, indicating an enhancement of fiber bridging ability existing in a higher fly ash system. (2) A lower matrix fracture toughness, which can be achieved by increasing the fly ash content, is considered an effective approach to enhancing the tensile strain capacity of ECC according to the micromechanics-based design theory for fiber-reinforced cementitious composites [11,40]. (3) Fly ash with the nature of spherical morphology functions in alleviating cutting and abrasion of fibers by the matrix, and theoretically, the above protective effect of fly ash is more significant for PVA fibers rather than PE fibers due to a lower elastic modulus of PVA fibers. Figure 7 illustrates that the promotion of the fibers’ protrusion length of FA50-A2-E0 versus FA30-A2-E0 was superior to that of FA50-A0-E2 versus FA30-A0-E2.

Compared to ECCs reinforced with sole PVA/PE fibers, ECCs incorporating hybrid PVA–PE fibers retained the high tensile strength associated with PE fibers while exhibiting enhanced tensile deformability. In this study, ECC incorporating 1% PVA and 1% PE fibers exhibited relatively high tensile strain capacity within the investigated mixtures, with ultimate tensile strength of 6.2 MPa and 4.5 MPa, and ultimate tensile strain of 3.2% and 5.3% for FA30-A1-E1 and FA50-A1-E1, respectively. The tensile strain capacity of 5.3% obtained in this study falls within the range reported for ECCs incorporating local fibers [1,20,21,22], indicating that hybrid local fibers can provide a level of tensile deformability comparable to that reported in previous studies. Given the significant enhancement provided by hybrid fibers, the next section further discusses the hybrid fiber effect and the mechanism.

#### 3.2.2. Hybrid Fiber Effect

**Figure 8:**
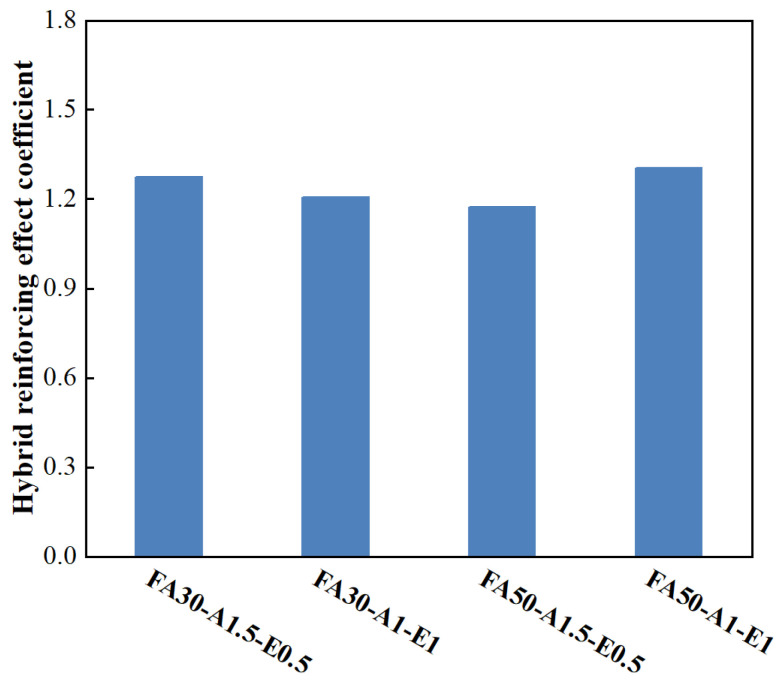
Hybrid reinforcing effect coefficient of ECCs containing hybrid fibers.

For a numerical comparison of the hybrid fiber effect on ECC’s deformation, the calculation of the hybrid reinforcing effect coefficient given in the previous reference was employed, as shown in Equation (7), which was accepted by relevant researchers in the field [41,42,43]. As shown in Figure 8, all ECCs containing hybrid fibers demonstrated positive hybrid effect, with the hybrid reinforcing effect coefficient of FA30-A1.5-E0.5, FA30-A1-E1, FA50-A1.5-E0.5, and FA50-A1-E1 being 1.27, 1.21, 1.17, and 1.30, respectively. According to the definition of the hybrid reinforcing effect coefficient, all values were greater than zero, indicating that positive hybrid reinforcing effects were achieved in all ECC mixtures containing hybrid fibers.

Although the differences among the calculated coefficients were relatively small and did not necessarily indicate statistically significant differences among the investigated mixtures, the consistently positive values suggest that the combined use of PVA and PE fibers is beneficial to the deformation performance of ECC. The positive hybrid effect may be attributed to the complementary roles of the two fibers. PVA fibers, with stronger fiber–matrix bonding, help promote stress transfer and crack distribution, whereas PE fibers, owing to their higher elastic modulus and pullout-dominated behavior, provide sustained fiber-bridging capacity during crack propagation. Furthermore, the positive hybrid reinforcing effect coefficients are in agreement with the synergistic behavior commonly observed in hybrid-fiber-reinforced systems reported in the literature [41,42,43]. Therefore, Figure 8 mainly serves to verify the existence of a positive hybrid reinforcing effect rather than to establish statistically significant differences among different hybrid fiber combinations.

(7) $$R=\frac{S-\sum_{1}^{n}S_{i}\varphi_{i}}{\sum_{1}^{n}S_{i}\varphi_{i}}$$

where *R* represents hybrid reinforcing effect coefficient, when *R* > 0, it means a positive hybrid effect, in contrast to when *R* < 0, it means a negative hybrid effect; *S* and *S_i_* represent the performance index of cementitious composites containing hybrid fibers and single fibers, respectively; and *φ_i_* = *V_i_*/*V*, *V_i_* and *V* represent the volume of a single fiber (named *i*-type) and hybrid fibers, respectively, with ∑*φ_i_* = 1.

**Figure 9:**
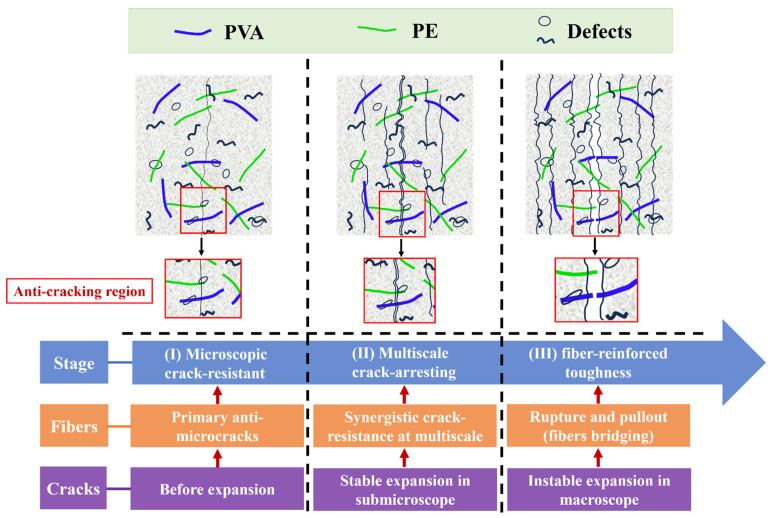
Conceptual progressive cracking mechanism of ECC reinforced with hybrid PVA–PE fibers, developed based on experimental observations obtained in this study and inspired by the conceptual framework reported in Ref. [44].

Referring to previous studies on the progressive crack resistance mechanism of hybrid fiber-reinforced cementitious composites incorporating different fibers [44], and combining the experimental observations obtained in this study, a conceptual progressive cracking mechanism is proposed for ECC reinforced with hybrid PVA–PE fibers of different elastic moduli, as illustrated in Figure 9. It should be noted that this mechanism is inferred from macroscopic cracking behavior, fiber pullout characteristics, and evidence reported in the literature, rather than direct microscopic observations. Accordingly, the tensile process is conceptually divided into the following three stages according to the crack-arresting effects of PVA and PE fibers during crack development.

(1) Stage I (microscopic crack-resistant stage): Stress redistribution occurs in ECC under tensile loading before crack expansion. Stress concentration at defects (e.g., voids and interfacial areas) will result in microcracks, but the bridging effect of the fibers prevents the expansion of these cracks.
(2) Stage II (multiscale crack-arresting stage): As tension increases, microscopic cracks grow into visible macroscopic cracks. PVA/PE fibers provide multiscale crack resistance, limiting further crack widening and expansion.
(3) Stage III (fiber-reinforced toughness stage): There are two fiber failure modes, i.e., pullout and rupture failure due to different properties of fibers. Subsequently, the stress redistribution occurs again, leading to continued fiber damage near the failed fiber region, and the crack advances through matrix fracture and multi-point fibrous failure.

In Table 5, compared to ECC containing single PVA/PE fibers, the extra gain in tensile strain of ECC incorporating hybrid fibers in equivalent amounts was 191% and 78% at low fly ash content, respectively, while they were 130% and 130% at high fly ash content. The results showed that PE fibers provided significant benefits in the low fly ash system, whereas the contribution of PVA/PE fibers in the high fly ash system was equally distributed. The reasons contributing to this situation are concluded as follows: On the one hand, the relatively higher fracture toughness of the low-fly ash matrix favors the role of PE fibers, whose high elastic modulus enables more effective fiber bridging and stress transfer during crack propagation. On the other hand, there is a strong interfacial bonding between the hydrophilic PVA surface and the matrix, and its low elastic modulus tends to dissipate a large amount of energy through rupture damage. With increasing fly ash content, the reduction in chemical bonding between PVA fibers and the matrix promotes fiber pullout rather than premature rupture, thereby allowing PVA fibers to contribute more effectively to tensile deformation. Therefore, the contribution of PVA fibers became increasingly significant in the high fly ash system.

#### 3.2.3. Cracking Patterns

**Figure 10:**
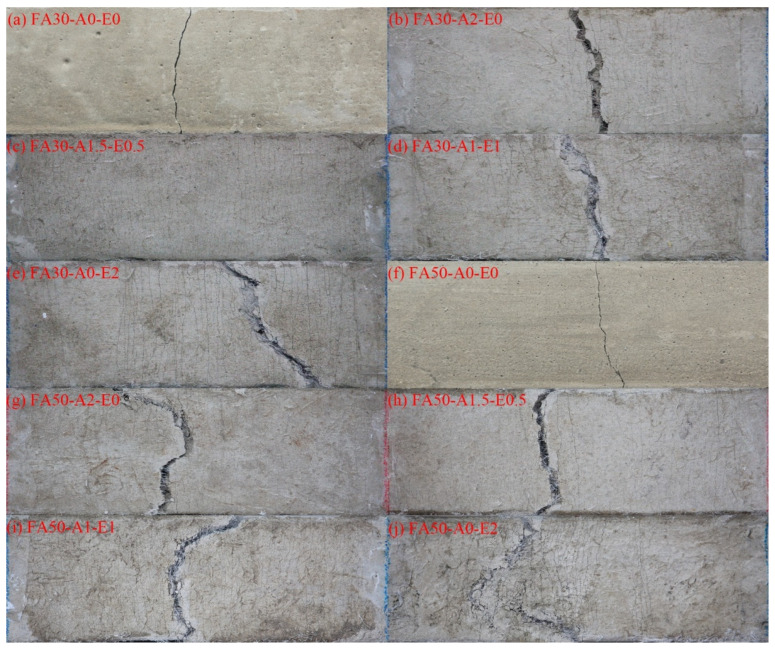
Crack failure patterns of ECCs and matrices.

Table 6 and Figure 10 show the cracking parameters and cracking failure patterns of ECCs and the corresponding matrices, respectively. The failed surface of tensile samples of ECCs exhibited multi-fine cracking characteristics, while the corresponding matrix was only accompanied by a single crack. In ECCs with 30% fly ash, PE fibers showed slightly better crack control than PVA fibers, i.e., a higher average number of cracks and a lower crack width. This behavior can be attributed to the relatively stronger chemical bonding between PVA fibers and the matrix at lower fly ash contents, which increases the likelihood of premature fiber rupture during crack propagation. In contrast, PE fibers mainly rely on frictional bonding and pullout mechanisms, enabling them to preserve fiber-bridging capacity more effectively under this condition. However, the crack-bridging role of PVA fibers became increasingly significant with increasing fly ash content. This behavior can be attributed to the reduced chemical bonding between the PVA fibers and the matrix (Table 4), resulting from the higher fly ash volume, which promoted fiber pullout rather than premature fiber rupture. Moreover, the spherical shape of fly ash may diminish the damage of PVA fibers during the pullout process, and this is why several studies have been conducted to enhance the tensile behaviors of ECC by surface modification of PVA fibers, such as oiling treatments [32].

Compared with ECCs reinforced with single fibers, ECCs incorporating hybrid fibers generally exhibited improved crack-control performance. FA50-A1-E1, which exhibited comparatively high tensile deformation capacity, showed an average crack number of 71, while FA30-A1.5-E0.5 exhibited the smallest average crack width of 39 μm among the mixtures investigated. PVA fibers contribute to stress transfer through stronger fiber–matrix bonding, whereas PE fibers provide sustained fiber-bridging capacity owing to their high elastic modulus and pullout-dominated behavior. The cracking parameters were generally consistent with the tensile results presented in Section 3.2.1. ECC mixtures exhibiting a larger number of cracks together with relatively smaller crack widths generally achieved superior tensile deformation capacity. This behavior indicates that tensile strain was distributed more uniformly throughout the specimen through the formation of multiple fine cracks rather than being concentrated into a few dominant cracks. Therefore, improved tensile performance is generally associated with the development of a greater number of tightly controlled cracks, which indicates enhanced strain distribution and more effective crack-bridging action of the fibers. The minimum average crack width of 39 μm achieved in this study falls within the range commonly reported for ECCs with multiple-cracking behavior [1,20,21,22], indicating effective crack-width control under tensile loading.

### 3.3. Compressive Strength

**Figure 11:**
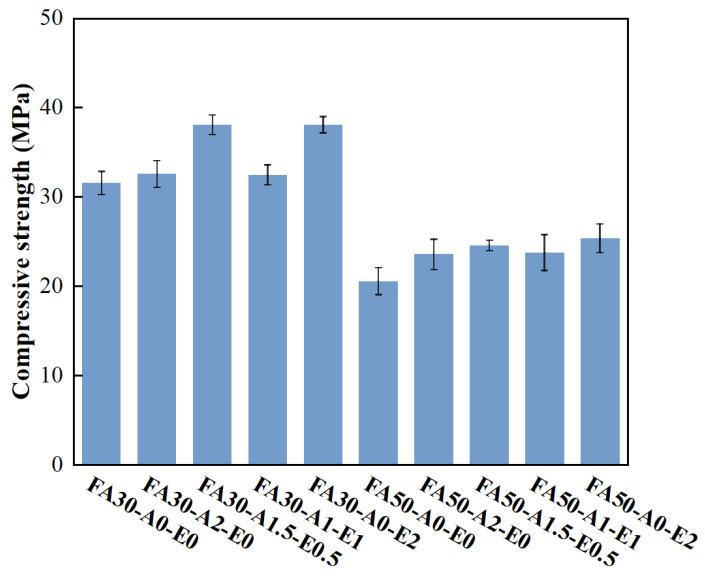
Compressive strength of ECCs and matrices.

In Figure 11, the compressive strength of the matrix decreases from 31.6 MPa to 20.6 MPa when the fly ash volume increases from 30% to 50%. This is attributed to the fact that the hydrating activity of fly ash is much lower than that of cement, and excessive amounts of fly ash mean that the cementitious proportion in raw materials is reduced, which is not conducive to the hydration and the increase in compressive strength. Specific density and porosity were not measured in this study; therefore, the influence of pore structure on compressive strength requires further investigation. Similarly, the aforementioned occurred in the ECCs. Comparing the results of compressive strength of ECCs and corresponding matrix, the fiber addition was beneficial to the development of compressive strength, attributable to the restraining effect of the fibers, which limits the horizontal expansion of ECC under compression [45,46]. It was notable that the compressive strength of ECC with hybrid PVA–PE fibers failed to surpass that of PVA/PE fibers’ single-addition, in which almost no change in strength was observed when the addition of PVA and PE fibers was 1% at the same time. FA50-A1-E1, which exhibited relatively high tensile deformation capacity among the investigated mixtures, demonstrated a compressive strength of 23.8 MPa. Considering the considerable variability in matrix composition and constituent materials among different ECC systems, the practical significance of directly comparing compressive strength values across studies is relatively limited. However, the compressive strength achieved in this study satisfies the requirements for non-structural repair materials [47,48]. Therefore, the ECCs incorporating hybrid local fibers developed in this study are promising candidates for engineering applications. Nevertheless, durability-related properties, including shrinkage, long-term performance, freeze–thaw resistance, and chloride penetration resistance, were beyond the scope of the present study and should be investigated in future work before broader practical application.

## 4. Conclusions

In this study, the pullout behaviors of single local PVA/PE fibers were investigated. The tensile properties and compressive strength of ECC incorporating fibers with various composition patterns, such as single-addition of PVA/PE fibers and co-addition of PVA–PE fibers, were further investigated. Based on the experimental results, the main conclusions are summarized as follows.

(1) The ECC with PVA–PE fibers co-addition generally exhibited improved tensile properties compared with ECC reinforced with single PVA or PE fibers, with a tensile strain of above 5.3% and an average crack width of below 39 μm, attributable to the hybrid fiber effect endowing diverse crack resistance. The hybrid reinforcing effect coefficients (*R*) of ECC containing hybrid fibers in this study were all over 0, ranging from 1.17 to 1.30, indicating a positive hybrid effect.
(2) The hydrophilic PVA fibers and hydrophobic PE fibers predominantly failed through rupture and pullout, respectively. The chemical bonding between PVA fiber and the surrounding matrix was reduced by increasing the volume of fly ash, and its fiber-bridging capacity was enhanced, as indicated by additional fiber protrusion at the fracture cross-section of ECC.
(3) The compressive strength of ECCs benefiting from the restraining effect of fibers was greater than that of the corresponding matrix, and the compressive strength of ECCs containing hybrid fibers failed to surpass that of ECCs reinforced with single fibers. The ECC with hybrid fibers in this study demonstrated a tensile strain of over 5.3% but a compressive strength not exceeding 28 MPa.

## Figures and Tables

**Table 1:** Chemical compositions of HBSAC and fly ash (wt%).

Types	CaO	SiO_2_	Al_2_O_3_	Fe_2_O_3_	MgO	Ti_2_O	SO_3_	K_2_O	LOI
HBSAC	35.45	10.67	11.09	1.43	2.11	0.18	10.34	–	27.89
Fly ash	10.11	38.48	28.99	10.24	2.56	–	1.98	2.20	–

**Table 2:** Properties of local PVA and PE fibers.

Fiber Type	Density (g/cm^3^)	Tensile Strength (MPa)	Elastic Modulus (GPa)	Elongation (%)	Diameter (μm)	Length (mm)
PVA	1.30	1300	35	7.5	39	12
PE	0.97	3000	110	2.0–3.0	24	12

**Table 3:** Mix proportions of ECC mixtures and corresponding matrices.

Mix ID	Binder Materials			Common Component (%)							Fibers (%)	
	HBSAC	FA	SF	SS	W	REP	TA	BA	AEA	SP	PVA	PE
FA30-A0-E0	68	30	2	58	30	0.77	0.20	0.21	0.02	0.20	0	0
FA30-A2-E0											2.0	0
FA30-A1.5-E0.5											1.5	0.5
FA30-A1-E1											1.0	1.0
FA30-A0-E2											0	2.0
FA50-A0-E0	48	50	2								0	0
FA50-A2-E0											2.0	0
FA50-A1.5-E0.5											1.5	0.5
FA50-A1-E1											1.0	1.0
FA50-A0-E2											0	2.0

**Table 4:** Parameters of single-fiber pullout.

Mix ID	Peak Load (N)	Displacement Corresponding to Peak Load (mm)	*G*_d_ (J/m^2^)	*τ*_0_ (MPa)	*β*
FA30-PVA	0.94 ± 0.21	0.58 ± 0.05	0.61 ± 0.18	2.12 ± 0.24	0.11 ± 0.02
FA30-PE	0.46 ± 0.05	0.15 ± 0.02	–	–	–
FA50-PVA	0.99 ± 0.11	0.83 ± 0.17	0.43 ± 0.16	2.43 ± 0.38	0.10 ± 0.02
FA50-PE	0.48 ± 0.09	0.18 ± 0.02	–	–	–

**Table 5:** Tensile parameters of ECCs and matrices.

Mix ID	Ultimate Tensile Strength (MPa)	Ultimate Tensile Strain (%)
FA30-A0-E0	4.0 ± 0.2	0.017 ± 0.001
FA30-A2-E0	5.2 ± 0.4	1.1 ± 0.4
FA30-A1.5-E0.5	5.4 ± 0.4	2.9 ± 0.7
FA30-A1-E1	6.2 ± 0.8	3.2 ± 0.1
FA30-A0-E2	5.8 ± 0.4	1.8 ± 0.3
FA50-A0-E0	3.4 ± 0.2	0.014 ± 0.002
FA50-A2-E0	3.9 ± 0.6	2.3 ± 0.6
FA50-A1.5-E0.5	4.2 ± 0.7	5.0 ± 1.7
FA50-A1-E1	4.5 ± 0.6	5.3 ± 1.0
FA50-A0-E2	3.9 ± 0.2	2.3 ± 0.7

**Table 6:** Cracking parameters of ECCs and matrices.

Mix ID	Average Crack Number	Average Crack Width (μm)
FA30-A0-E0	1 ± 0	–
FA30-A2-E0	21 ± 1	77 ± 11
FA30-A1.5-E0.5	66 ± 8	39 ± 2
FA30-A1-E1	46 ± 3	46 ± 13
FA30-A0-E2	25 ± 3	67 ± 14
FA50-A0-E0	1 ± 0	–
FA50-A2-E0	36 ± 14	65 ± 12
FA50-A1.5-E0.5	51 ± 1	72 ± 2
FA50-A1-E1	71 ± 1	64 ± 14
FA50-A0-E2	27 ± 4	62 ± 10

## Data Availability

The original contributions presented in this study are included in the article. Further inquiries can be directed to the corresponding author.
